# Supplementary material for: Terephthalate Copolyesters Based on 2,3-Butanediol and Ethylene Glycol and Their Properties
Source: Polymers (Basel). 2024 Jul 30;16(15):2177. doi: 10.3390/polym16152177 (PMC11314993; doi:10.3390/polym16152177)
Supplement: Supplementary file 1 [file polymers-16-02177-s001.zip › polymers-3106261-supplementary.pdf]

Supplementary Material

Terephthalate copolyesters based on 2,3-butanediol and ethylene glycol and their properties

Marian Blom[1,2], Robert-Jan van Putten[1,2], Kevin van der Maas[2], Bing Wang[2], Gerard P. M. van Klink[1,2], and Gert-Jan M. Gruter [1,2,*]

1 Industrial Sustainable Chemistry, Universiteit van Amsterdam, Science Park 904, 1098 XH Amsterdam, The Netherlands; m.blom@uva.nl (M.B.); g.j.m.gruter@uva.nl (G.-J.M.G.)

2 Avantium N.V., Zekeringstraat 29, 1014 BV Amsterdam, The Netherlands;Affiliation 1; marian.blom@avantium.com (M.B.); kevin.vandermaas@avantium.com (K.v.d.M.); gerard.vanklink@avantium.com (G.P.M.K.); bing.wang@avantium.com (B.W.); robert-jan.vanputten@avantium.com (R.-J.v.P.) gert-jan.gruter@avantium.com (G.-J.M.G.)

* Correspondence: g.j.m.gruter@uva.nl

Short index

Figure S1.	Heating segments of both cycles (top set: cycle 1, bottom set: cycle 2) of polyesters synthesized at small scale in order of increasing 2,3-BDO content
Figure S2.	P23B(43)ET DSC
Figure S3.	DSC results RAMA N180 PET from Indorama and PETG from 123-3D BV.
Figure S4.	Spectra after step 1 (transesterification) before PC of P23BT.
Figure S5-S10.	NMR spectra of final polymers
Table S1.	Calculations % 2,3-BDO (excluding end groups) based on ^{1}H NMR data
Figure S11.	MBL119 P23B(43)ET liquid collected from cold trap
Graph S1-S6 and Table S2-S5.	Data extensometer of all measured polymers and their results
Table S7 and Graph S6.	Barrier results (including measurement at different temperatures and humidities)
Figure S12-S17.	GPC results of final polymers
Table S8.	Data impact tests

DSC results

Methods segments:

1. 25.0 °C, 5 min., N_2 50 ml/min
2. 25.0 → 300.0 °C, 10.00 K/min, N_2 50 ml/min
3. 300.0 → 25.0 °C, -10.00 K/min, N_2 50 ml/min
4. 25.0 °C, 5 min., N_2 50 ml/min
5. 25.0 → 300.0 °C, 10.00 K/min, N_2 50 ml/min
6. 300.0 → 25.0 °C, -40.00 K/min, N_2 50 ml/min

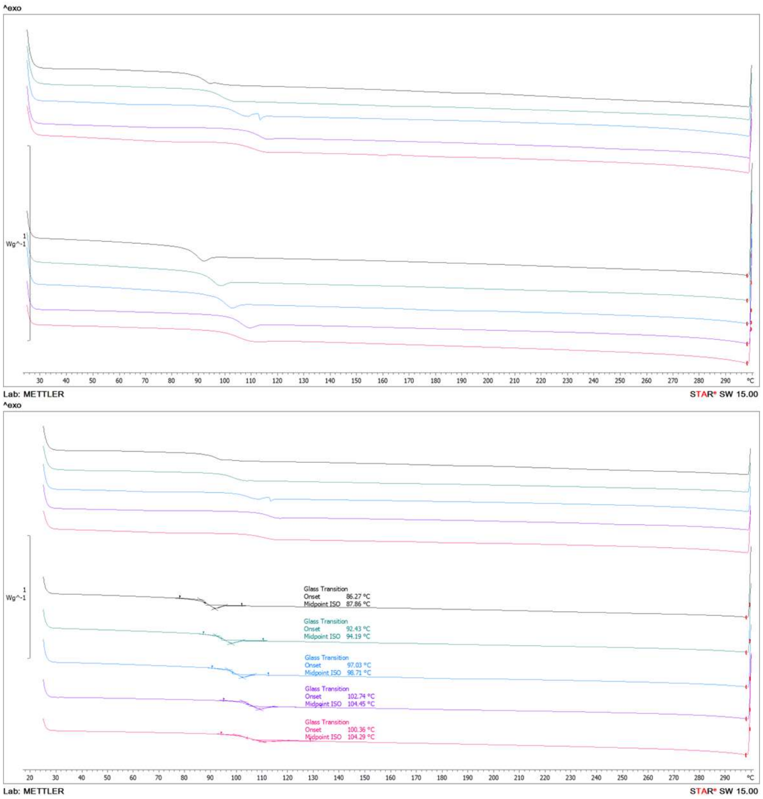

Figure S1. Heating segments of both cycles (top set: cycle 1, bottom set: cycle 2) of polyesters synthesized at small scale in order of increasing 2,3-BDO content (top to bottom per cycle). Tg taken from second cycle.

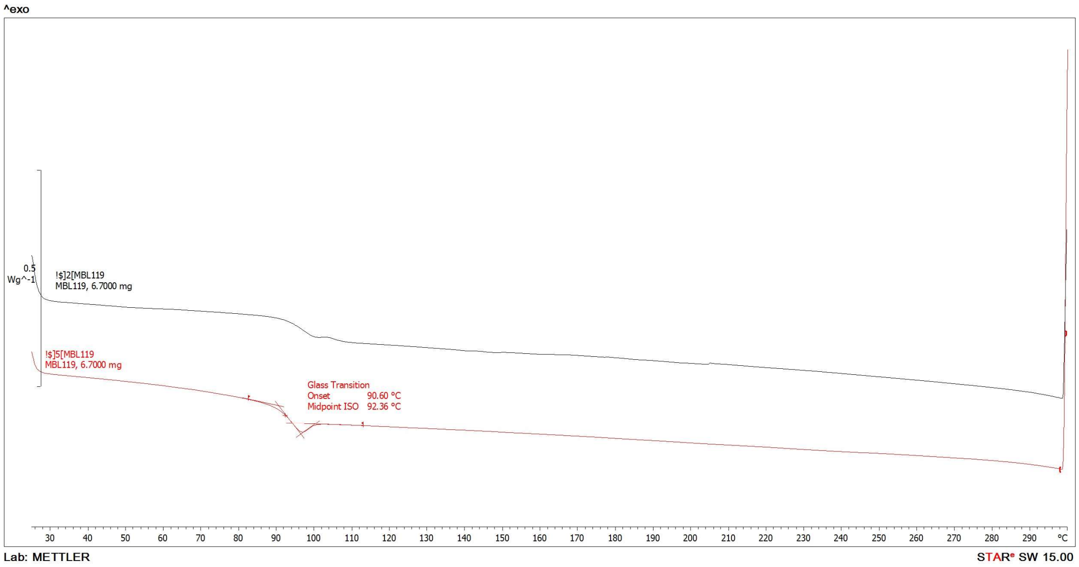

Figure S2. P23B(43)ET DSC

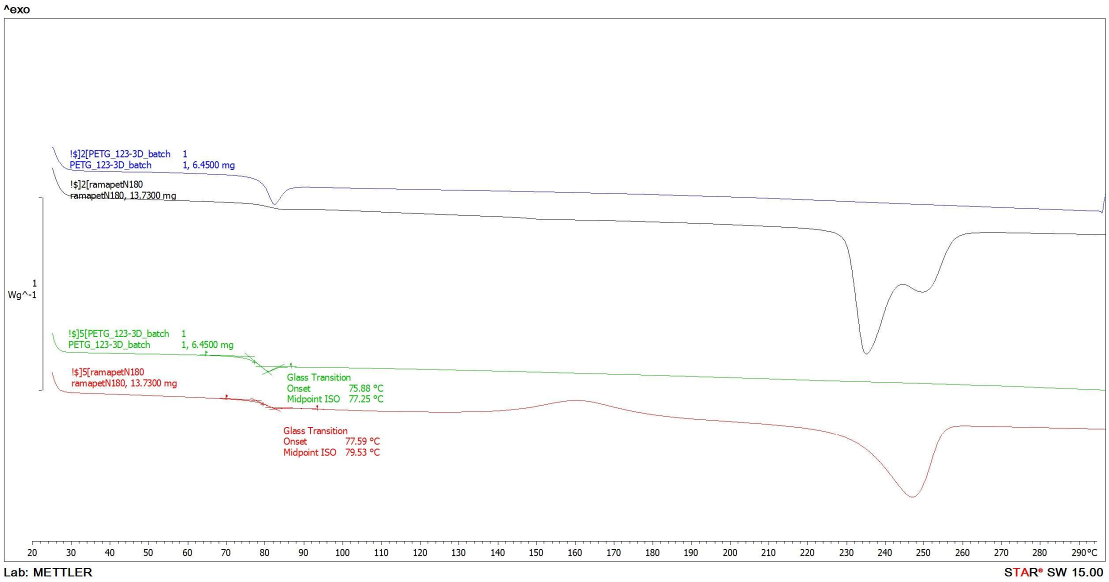

Figure S3. DSC results RAMA N180 PET from Indorama and PETG from 123-3D BV.

NMR Results

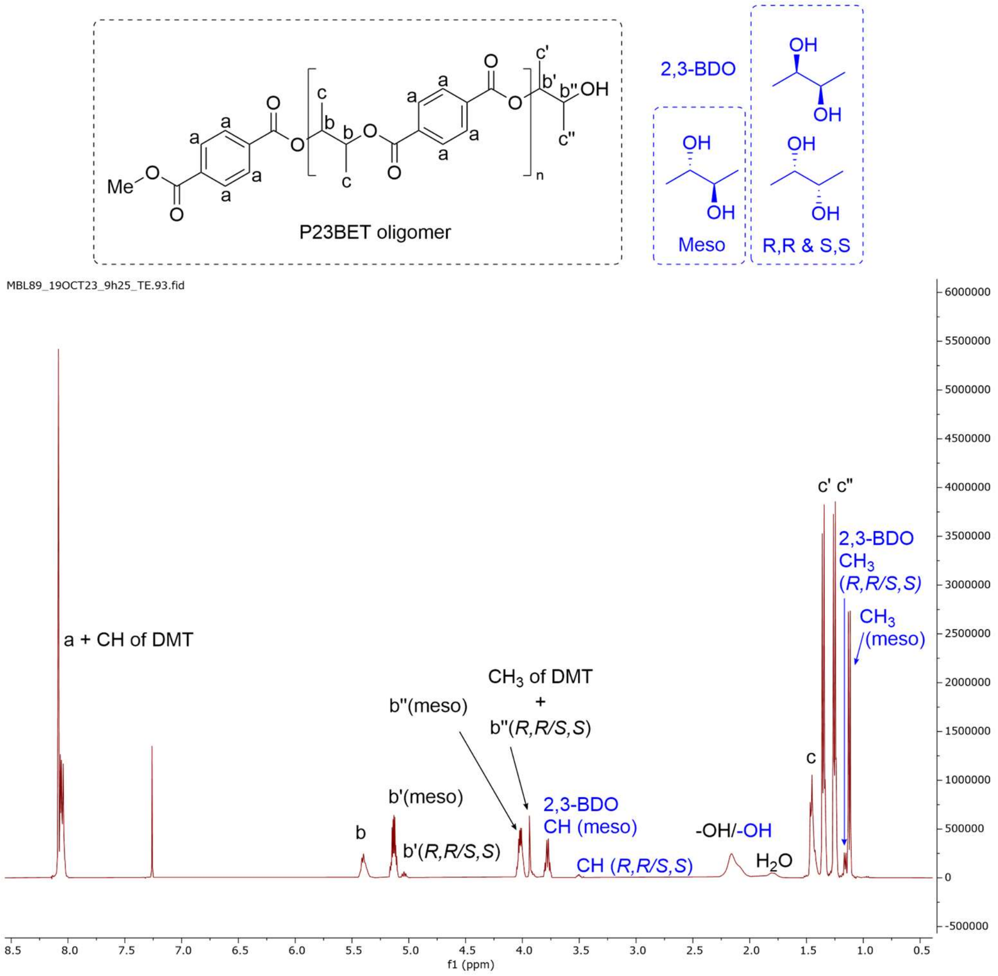

Figure S4. Spectra after step 1 (transesterification) before PC of P23BT.

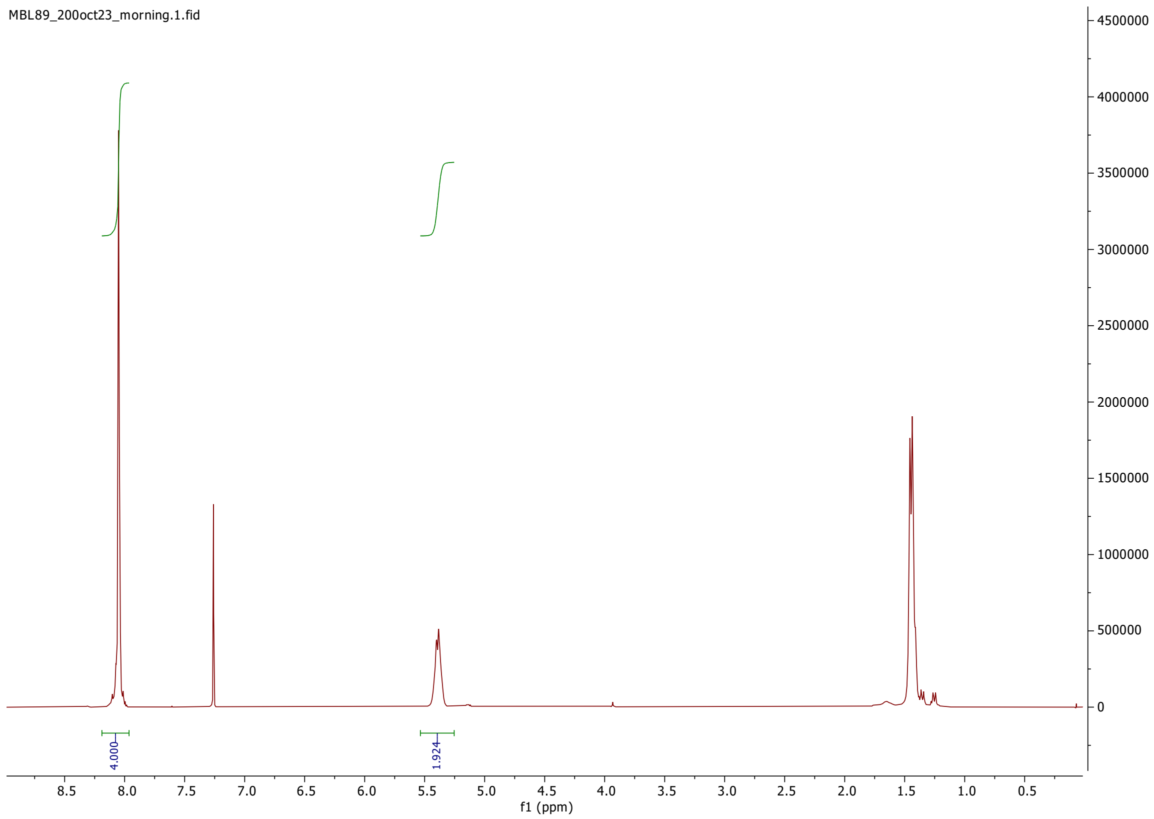

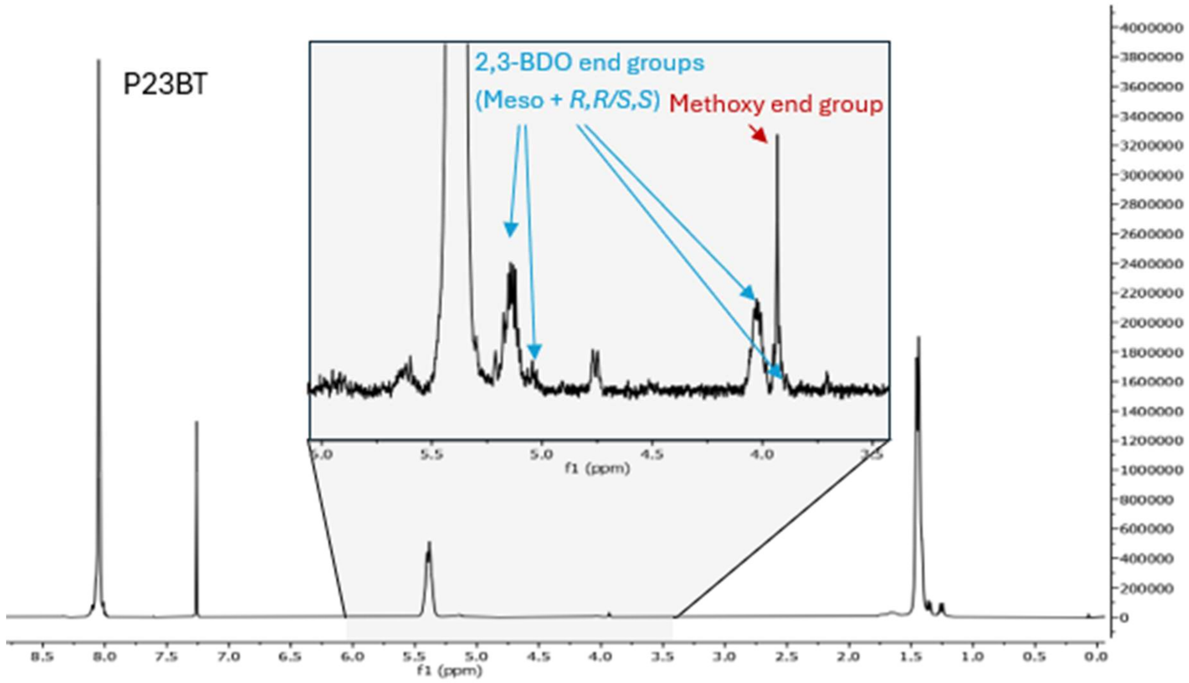

Figure S5. P23BT final polymer

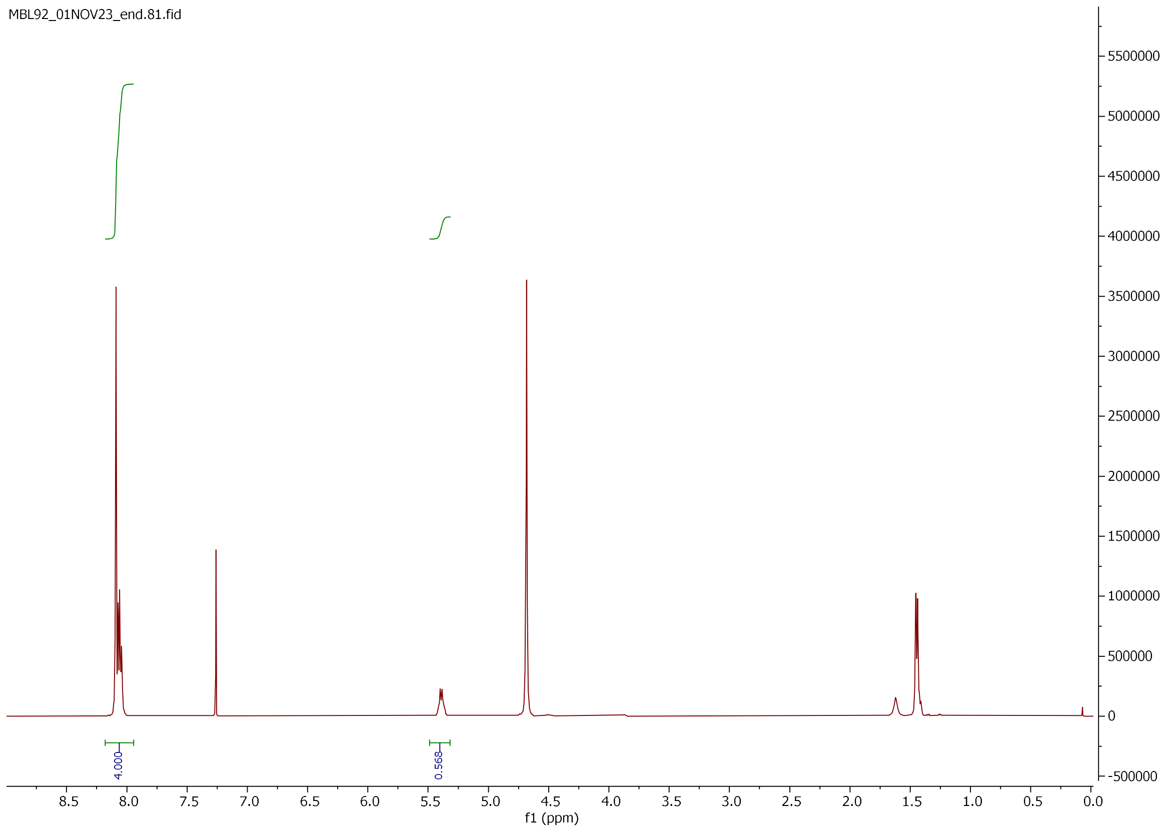

Figure S6. MBL92 P23B(28)ET final polymer

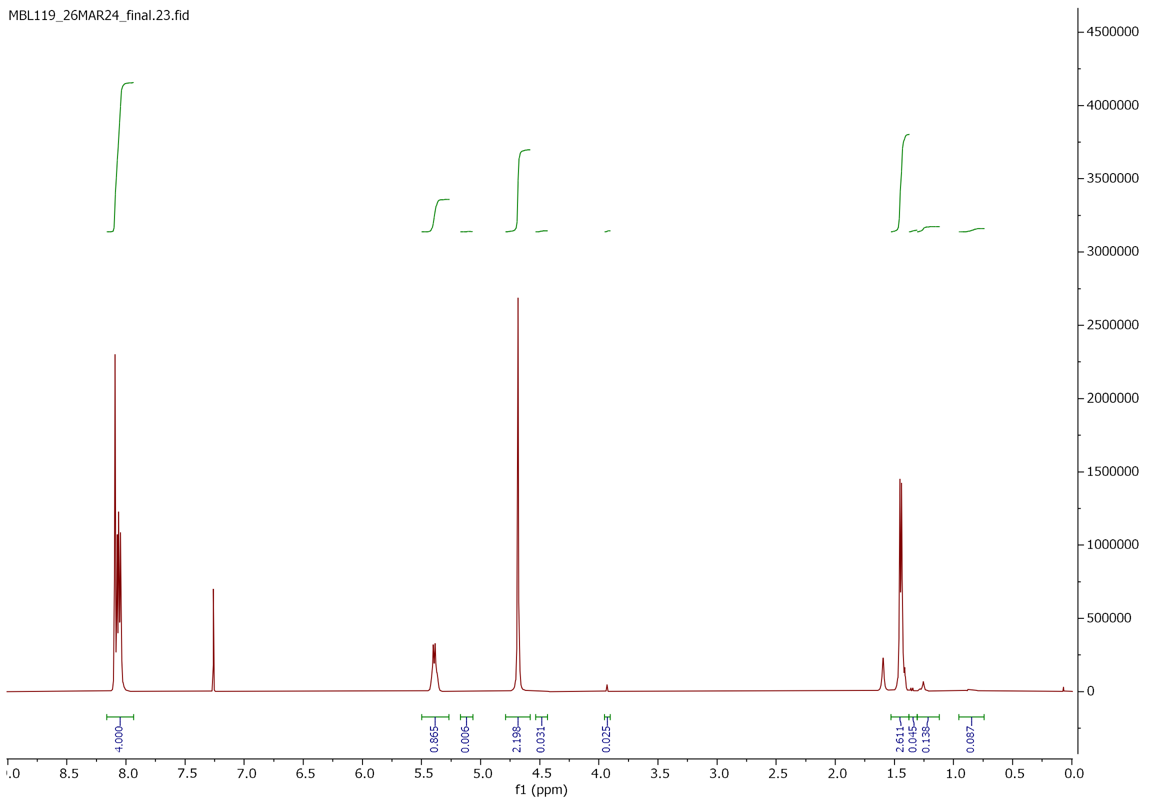

Figure S7. MBL119 P23B(43)ET final polymer

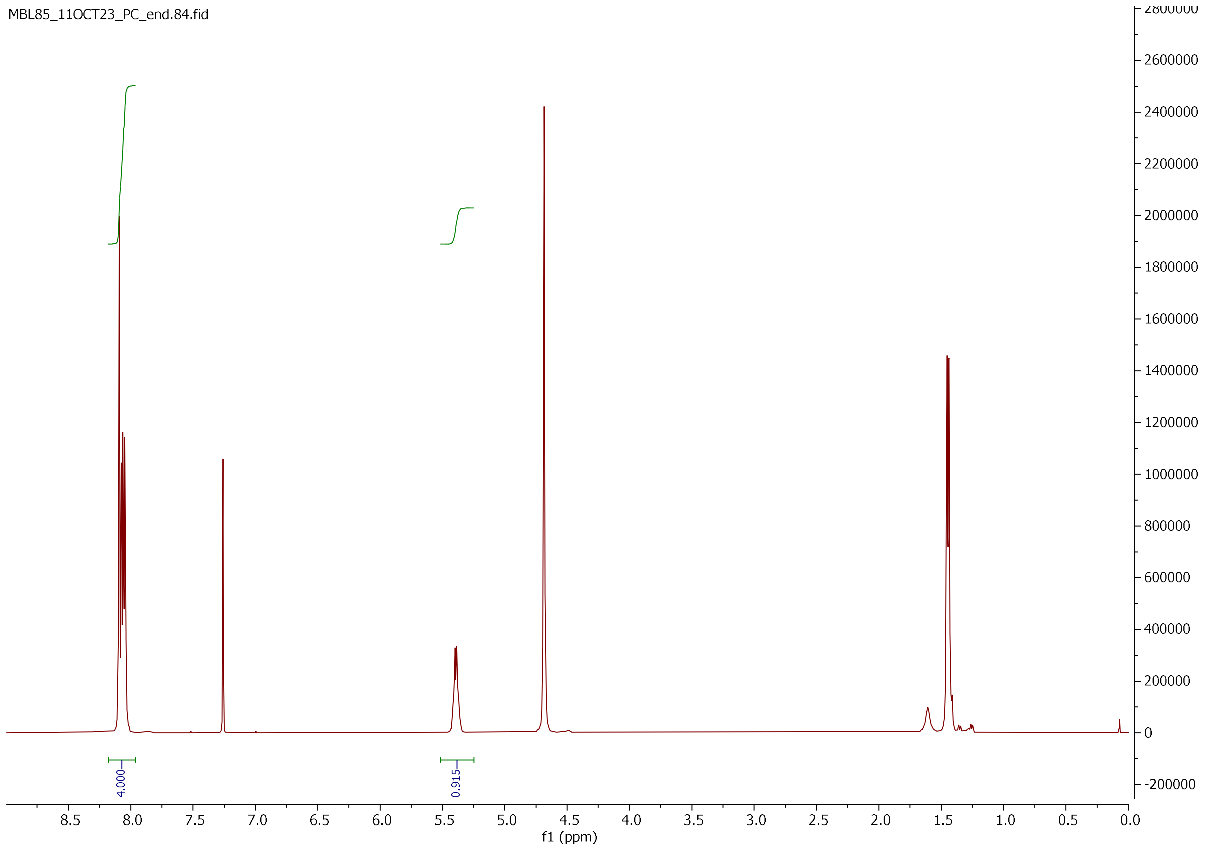

Figure S8. MBL85 P23B(46)ET final polymer

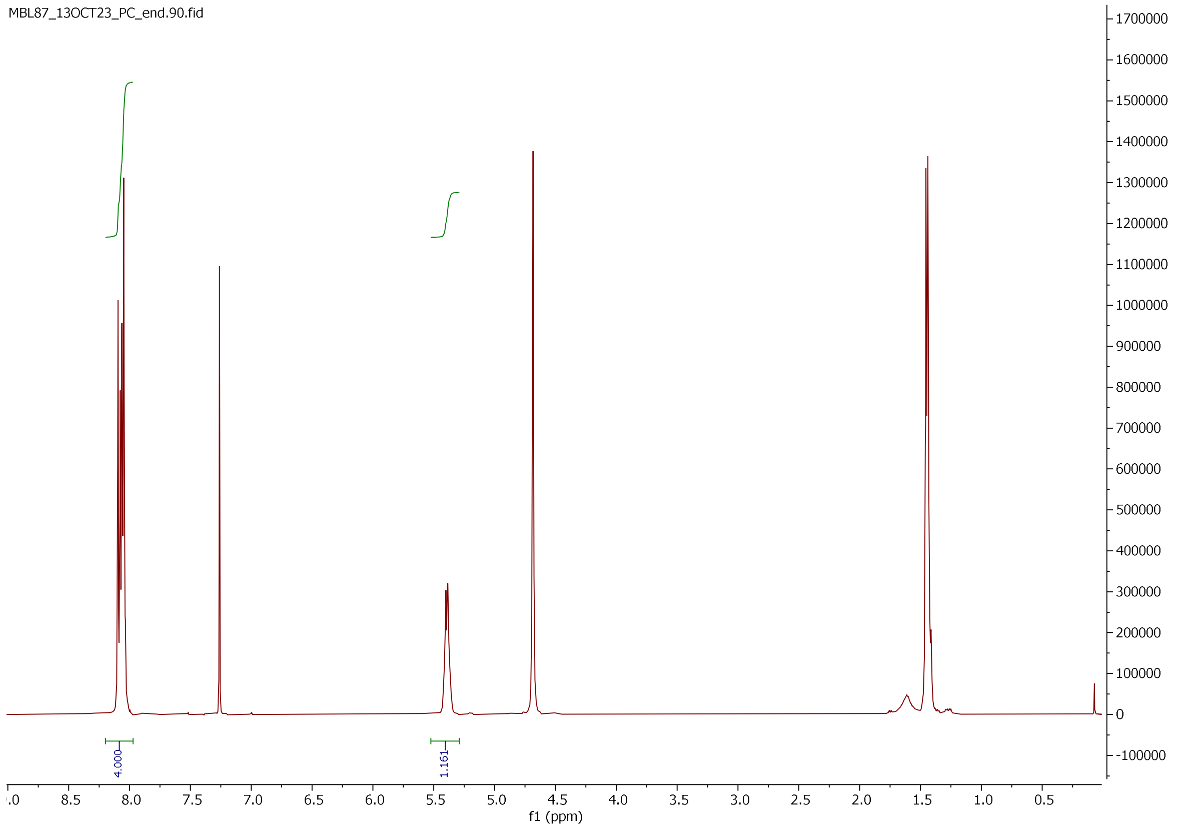

Figure S9. **MBL87 P23B(58)ET final polymer**

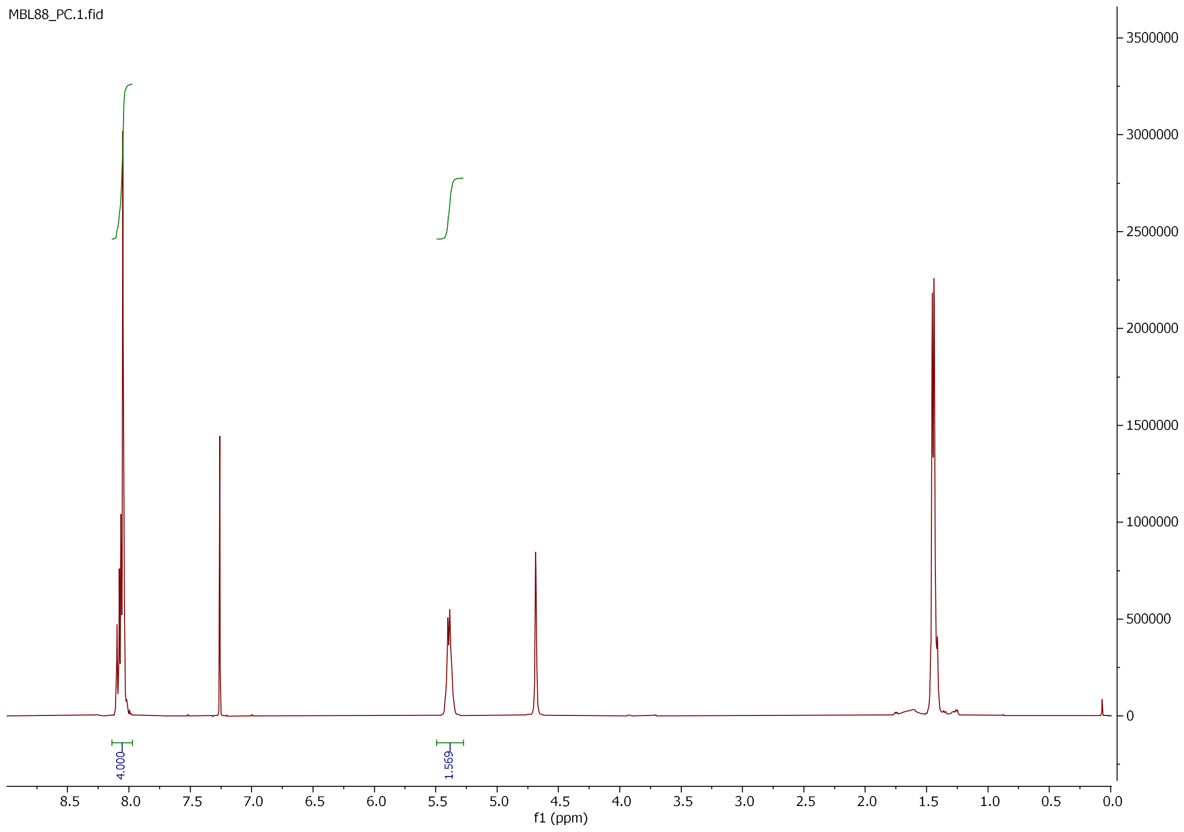

Figure S10. MBL88 P23B(78)ET final polymer

Table S1. Calculations % 2,3-BDO (excluding end groups) based on ^{1}H NMR data

integral a (8.08 ppm)	integral b (5.39 ppm)	Integral a / 4 H	Integral b / 2 H	P23B(%)ET
4.00	0.568	1	0.28	P23B(28)ET
4.00	0.865	1	0.43	P23B(43)ET
4.00	0.915	1	0.46	P23B(46)ET
4.00	1.161	1	0.58	P23B(58)ET
4.00	1.569	1	0.78	P23B(78)ET

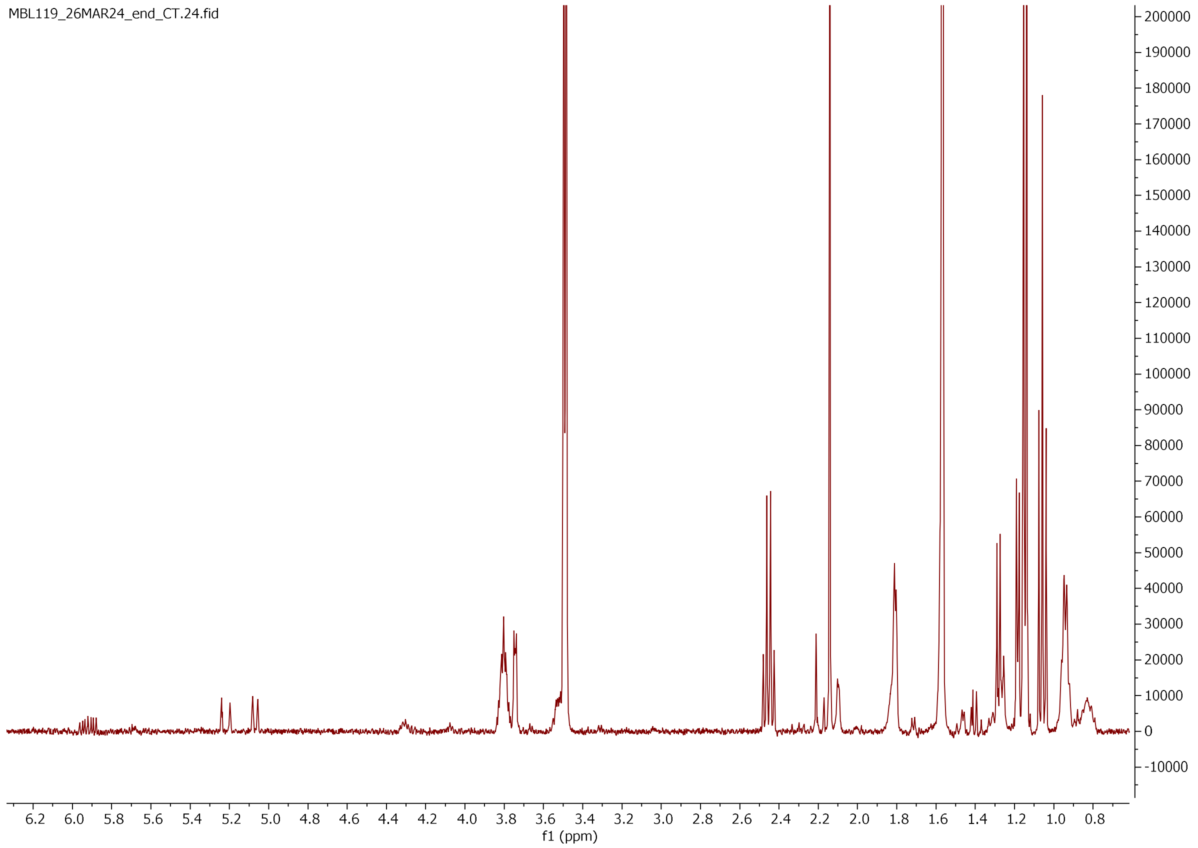

Figure S11. **MBL119 P23B(43)ET liquid collected from cold trap**

Tensile test results

Normalized strain at start to zero value and calculated slope on values between 0.0005 mm/mm strain and 0.0025 mm/mm for the modulus. Extension at break, max tensile stress and yield offset as calculated and/or provided by software.

Elongation % = “extension at break (mm)” / “Tensile bar length 20 (mm)” * 100%

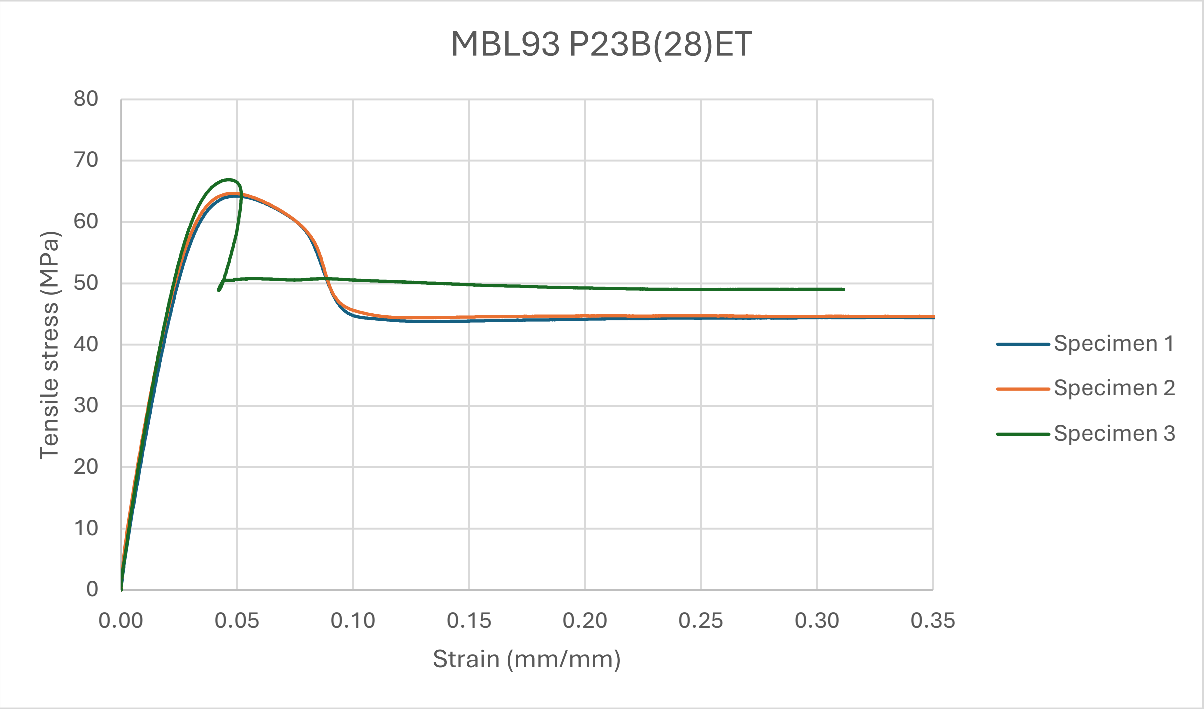

Graph S1. Data extensometer P23B(28)ET

Table S2. P23B(28)ET Tensile test results

MBL93 P23B(28)ET Specimen	Modulus (MPa)	Max. Tensile stress (MPa)	Extension at break (mm)	Elongation %	Tensile stress at Yield (Offset 0.002 mm/mm) (MPa)
1	2697	64.24	49.64	248.2	42.34
2	3254	64.67	73.52	367.6	37.96
3	2481	66.88	65.44	327.2	42.24
Average Specimen	2811	65.26333	62.86667	314.3333	40.85
Standard deviation	398.84	1.42	12.15	60.73	2.5

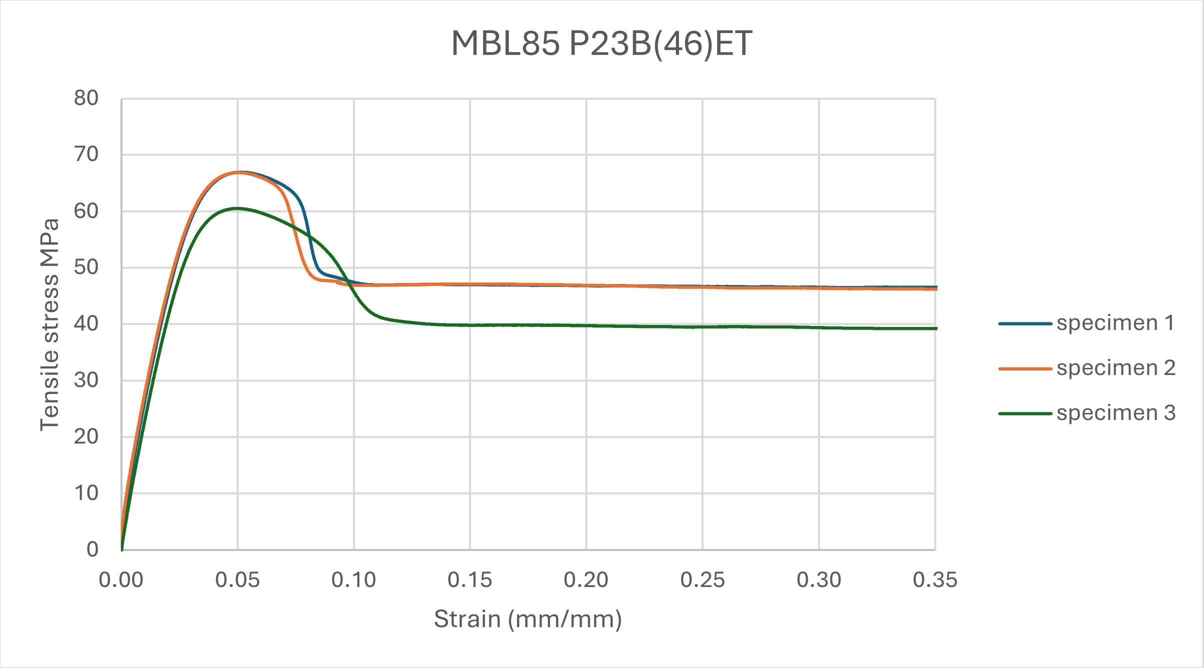

Graph S2. Data extensometer P23B(46)ET

Table S3. P23B(46)ET Tensile test results

MBL85 P23B(46)ET Specimen	Modulus (MPa)	Max. Tensile stress (MPa)	Extension at break (mm)	Elongation %	Tensile stress at Yield (Offset 0.002 mm/mm) (MPa)
1	2920.5	66.96	58.26	291.3	40.25
2	2966.4	66.88	51.94	259.7	38.85
3	2528	60.53	73.81	369.1	40.07
Average	2804.97	64.79	61.34	306.7	39.72
Standard deviation	240.96	3.69	11.25	56.30	38.85

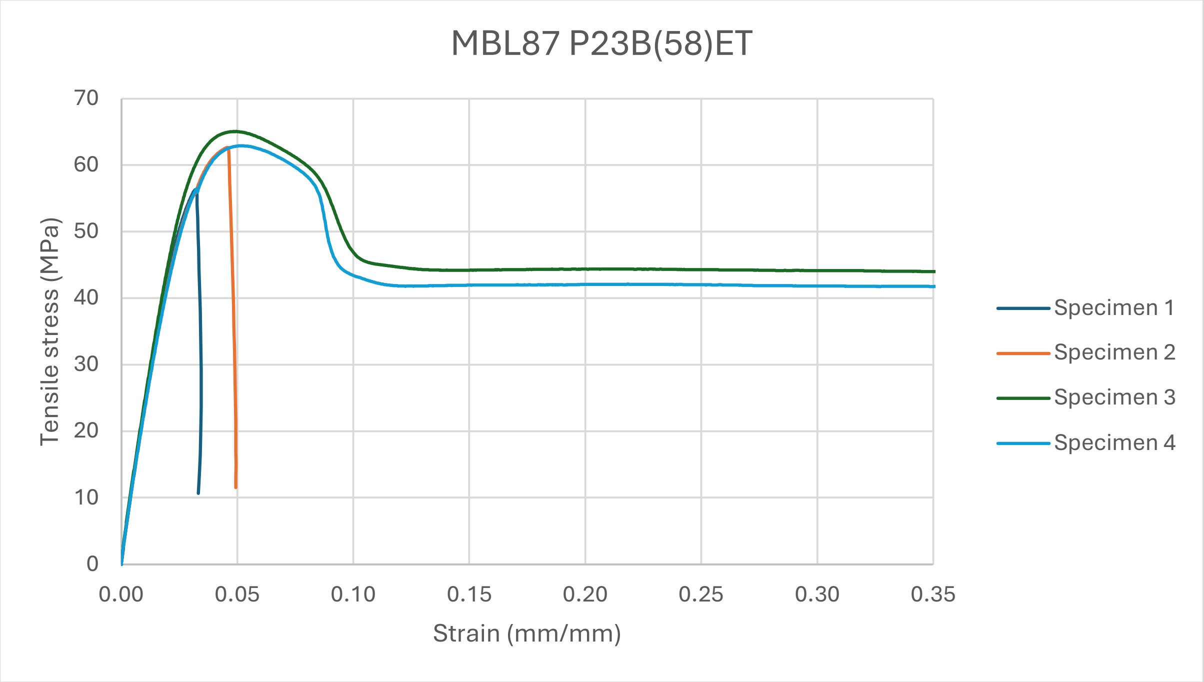

Graph S3. Data extensometer P23B(58)ET

Table S4. P23B(58)ET Tensile test results

MBL87 P23B(58)ET Specimen	Modulus (MPa)	Max. Tensile stress (MPa)	Extension at break (mm)	Elongation %	Tensile stress at Yield (Offset 0.002 mm/mm) (MPa)
1	2558.8	56.38	1.52	7.6	40.06
2	2554.5	62.64	2.02	10.1	41.02
3	2632	65.03	46.76	233.8	46.41
4	2543	62.89	46.78	233.9	41.37
Average	2572	61.735	24.27	121.35	42.22
Standard deviation	40.50	3.73	25.98	129.91	2.85

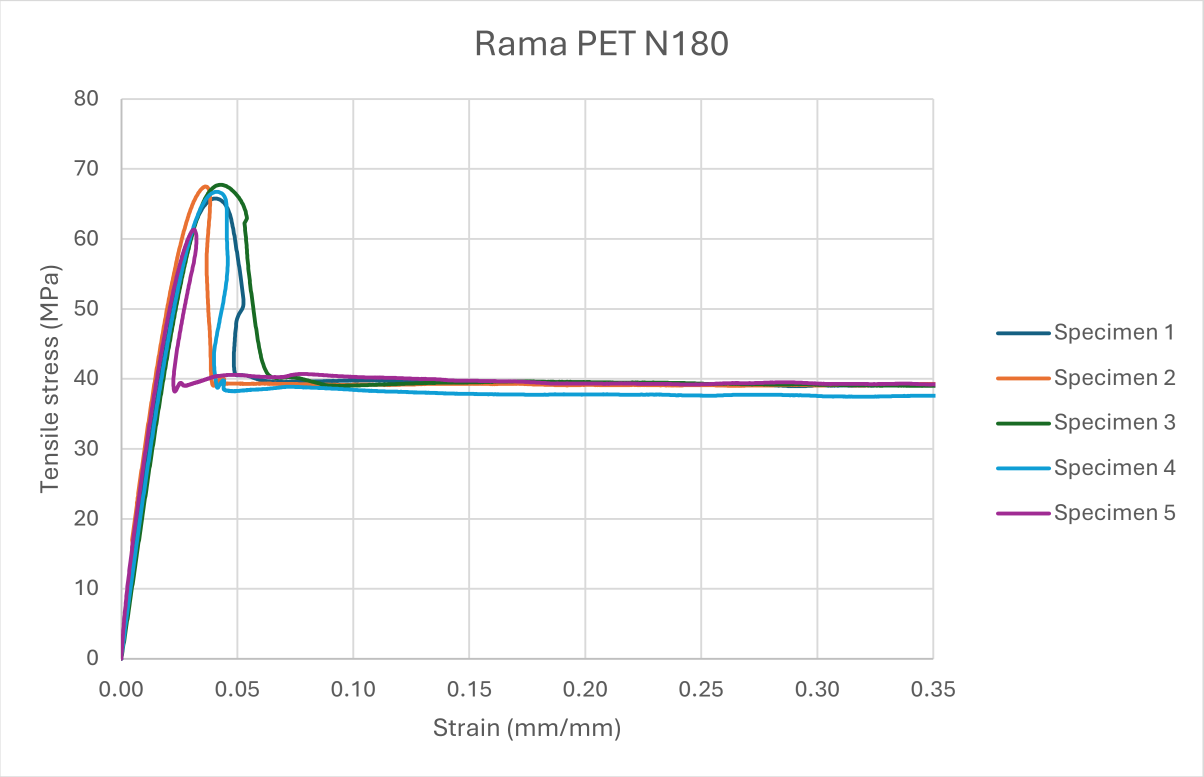

Graph S4. Data extensometer PET

Table S5. PET Tensile test results

PET Specimen	Modulus (MPa)	Max. Tensile stress (MPa)	Extension at break (mm)	Elongation %	Tensile stress at Yield (Offset 0.002 mm/mm) (MPa)
1	2781.49	65.77	99.84	328.85	43.30
2	3263.88	67.49	122.4	337.45	35.88
3	2165.15	67.74	117.35	338.70	54.85
4	2542.19	66.74	75*	375*	46.87
5	3630.39	61.34	85.57	306.70	33.94
Average	2876.62	65.82	100.03	337.34	42.97
Standard deviation	579.92	2.62	18.09	22.05	8.49

*no automatic log by the program, calculated based on fractions

Some deviation in processing:
Tensile specimen 1: 700 bar instead of 960 bar.
Tensile specimen 2: 510 bar instead of 960 bar.
Tensile specimen 3: 510 bar instead of 960 bar.
Tensile specimen 4: 440 bar instead of 960 bar.
Tensile specimen 5: no deviation from protocol.
Other tensile specimen and impact bars were processed at a pressure of 960 bar.

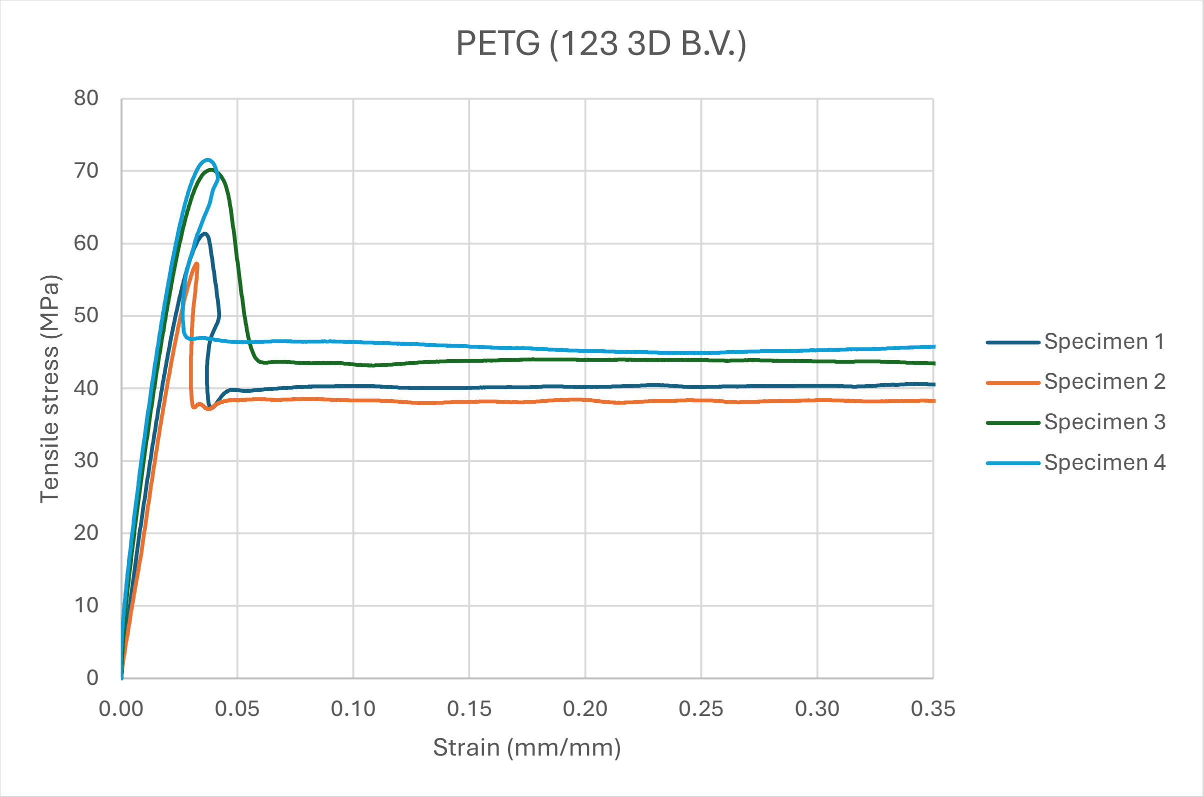

Graph S5. Data extensometer PETG

Table S6. PETG Tensile test results

PETG Specimen	Modulus (MPa)	Max. Tensile stress (MPa)	Extension at break (mm)	Elongation %	Tensile stress at Yield (Offset 0.002 mm/mm) (MPa)
1	2717.12	61.37	58.5*	292.5*	40.71
2	2084.13	57.25	60.69	303.45	51.63
3	3908.25	70.16	71.65	358.25	37.87
4	3400.10	71.54	64.75	323.75	38.15
Average	3027.40	65.08	63.90	319.49	42.09
Standard deviation	795.99	6.89	5.78	28.90	6.49

*no automatic log by the program, calculated based on fractions

Barrier results

Table S7. Barrier results

	thickness [mm]	Transmission @ 90% [g/(m2x24h)]	Transmission @ 100% [cm3/(m2x24h)]				WP [mmxg/(m2x24h)]/kPa	OP [mmxcm3/(m2x24h)]/bar			
Measurement		WV 90% hum. 38° C	O_2 50% hum. 30 °C	O_2 0% hum. 30 °C	O_2 50% hum. 23 °C	O_2 0% hum. 23 °C	38 °C	O_2, 50% hum. 30 °C	O_2, 0% hum. 30 °C	O_2, 50% hum. 23 °C	O_2, 0% hum. 23 °C
PET*	0.100	15.064[1]	34.978[1]				0.252[1]	3.5[1]			
PETG	0.132	12.125	79.361	85.235	63.803	68.619	0.268	10.5	11.3	8.4	9.1
P23B(28)ET	0.168	16.123	120.736	112.389	101.199	79.875	0.454	20.3	18.9	17.0	13.4
P23B(46)ET	0.120	28.153	236.783	255.842	197.649	211.645	0.566	28.4	30.7	23.7	25.4
P23B(58)ET	0.118	33.545	437.308	462.22	372.386	397.63	0.663	51.6	54.5	43.9	46.9
All values are corrected for film tickness. In addition, for the WP, the WV transmission is devided by 5.9697 to obtain "WP". (At 90% humidity the water saturation pressure is 6.0, to obtain WP, the WV transmisitoin rate is divided by 6 and corrected to kPa instead of bar) *PET values from published work.[1]											

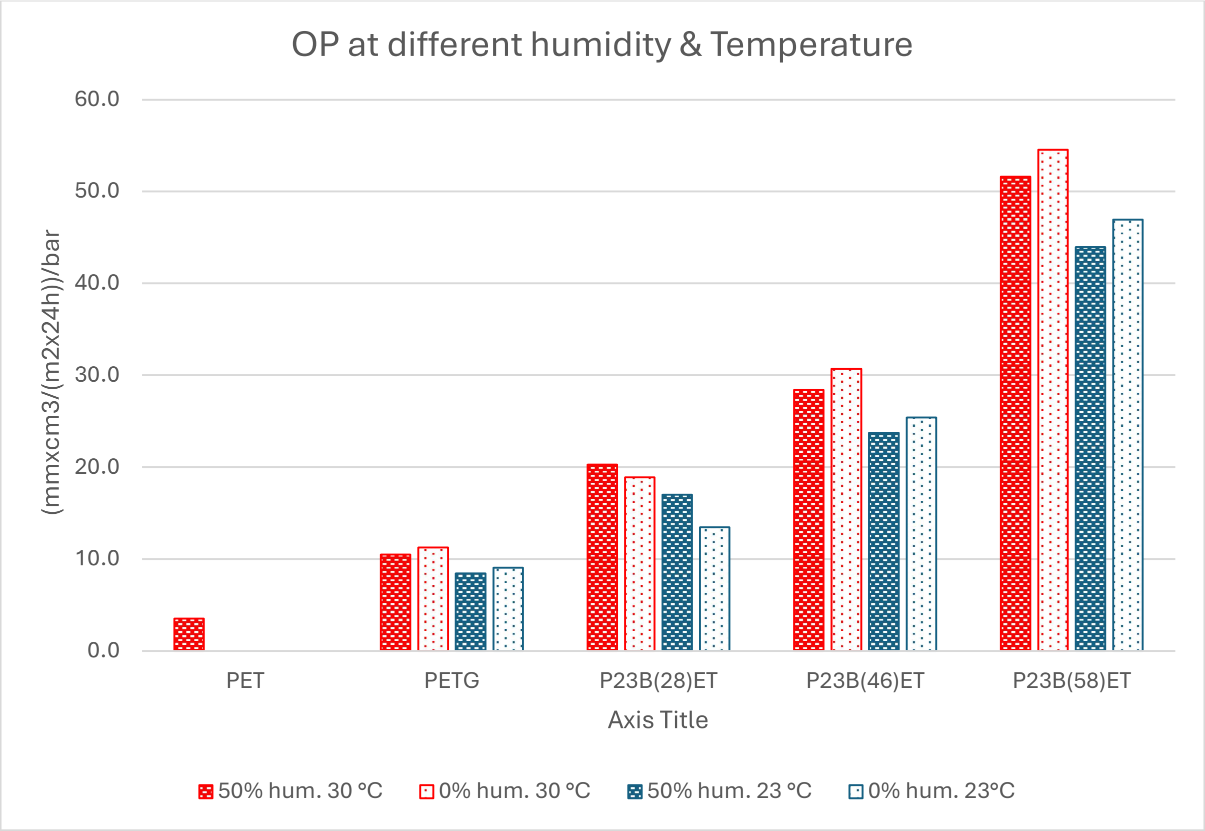

Graph S6. OP at different humidity and temperature

GPC Results

**Instrument:**	SP GPC	**Location:**	P1-F1
**Injection date:**	2023-11-01 15:37:38+01:00	**Injection:**	1 of 1
**Acq. method:**	GPC_DCM.amx	**Injection volume:**	50.000
**Analysis method:**	GPC_Standard processing method new calibration 11-Jan-23.pmx	**Acq. operator:**	SYSTEM
**Last changed:**	2023-01-11 15:55:11+01:00		

Peak #	RT (min)	Mn (g/mol)	Mw (g/mol)	PD	Mp (g/mol)
1	13.627	31508	75114	2.38	71844
2	17.393	1160	1165	1	1132
3	17.716	785	809	1.03	843

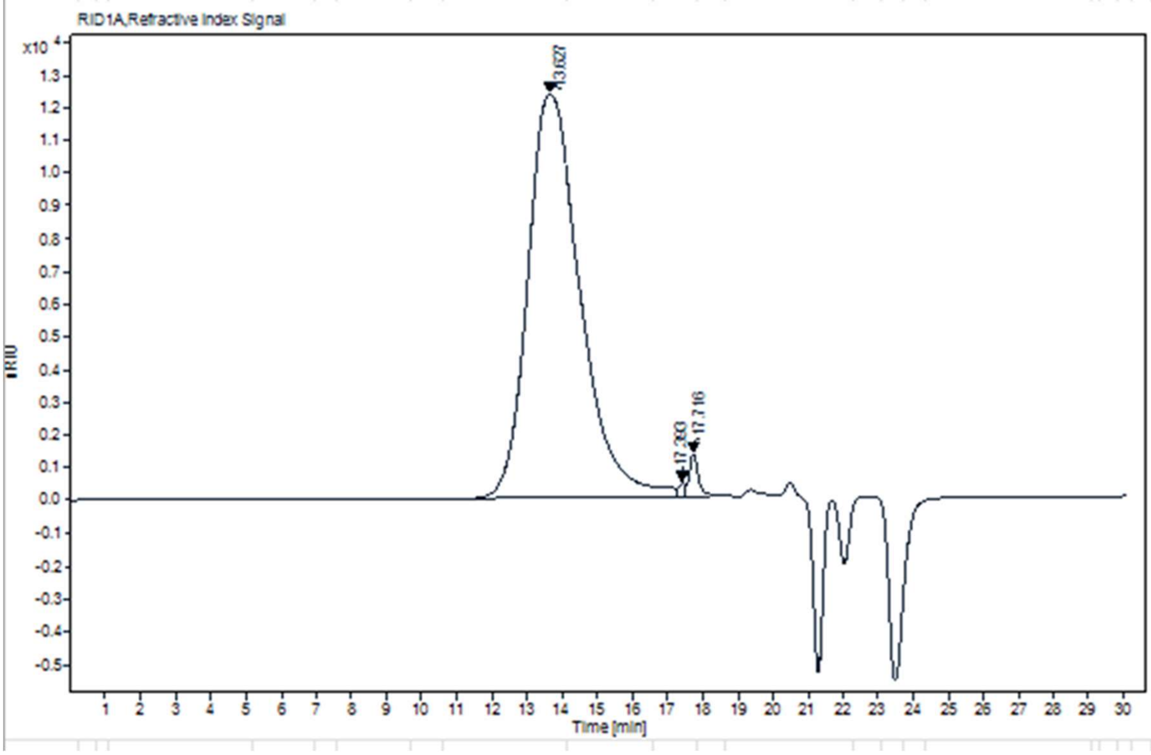

Figure S12. GPC results of P23B(28)ET

Instrument:	SP GPC	Location:	P1-E3
Injection date:	2023-10-18 15:27:08+02:00	Injection:	1 of 1
Acq. method:	GPC_DCM.amx	Injection volume:	50.000
Analysis method:	GPC_Standard processing method new calibration 11-Jan-23.pmx	Acq. operator:	SYSTEM
Last changed:	2023-01-11 15:55:11+01:00		

Peak #	RT (min)	Mn (g/mol)	Mw (g/mol)	PD	Mp (g/mol)
1	13.869	26556	59893	2.26	55250
2	17.358	1196	1201	1	1180
3	17.674	862	871	1.01	877

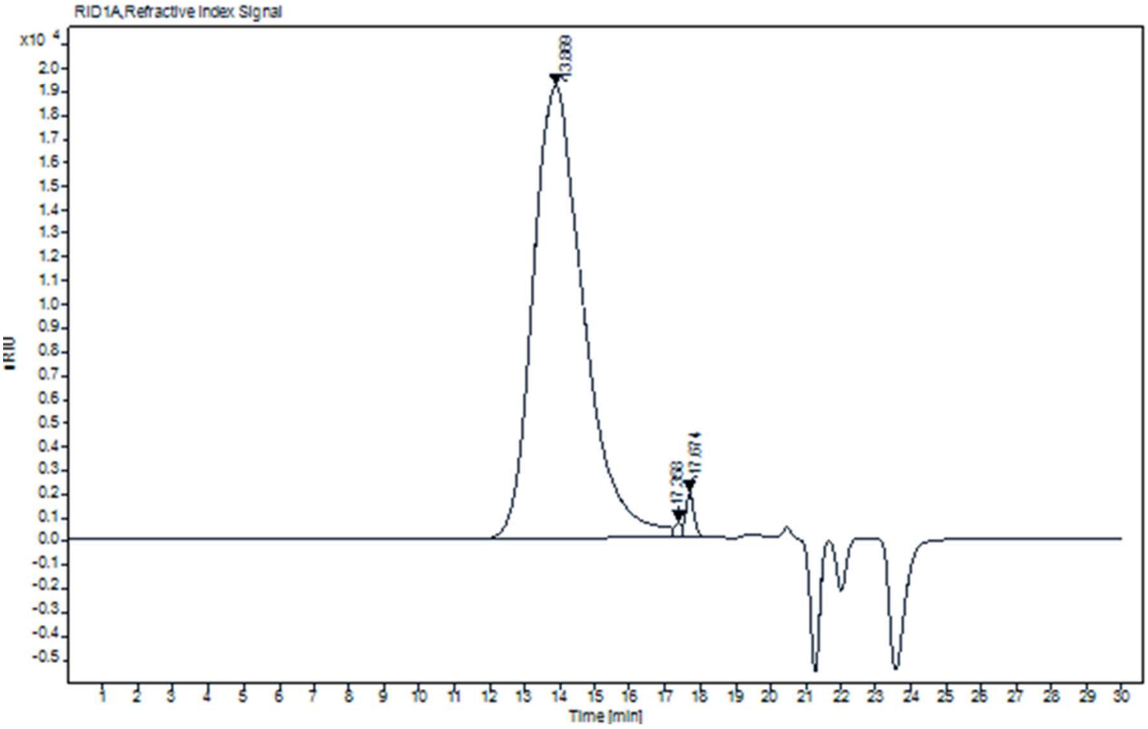

Figure S13. GPC results of P23B(46)ET

**Instrument:**	SP GPC	**Location:**	P1-E4
**Injection date:**	2023-10-16 16:47:49+02:00	**Injection:**	1 of 1
**Acq. method:**	GPC_DCM.amx	**Injection volume:**	50.000
**Analysis method:**	GPC_Standard processing method new calibration 11-Jan-23.pmx	**Acq. operator:**	SYSTEM
**Last changed:**	2023-01-11 15:55:11+01:00		

Peak #	RT (min)	Mn (g/mol)	Mw (g/mol)	PD	Mp (g/mol)
1	13.911	24639	56392	2.29	52149
2	17.324	1234	1240	1	1212
3	17.636	884	894	1.01	907

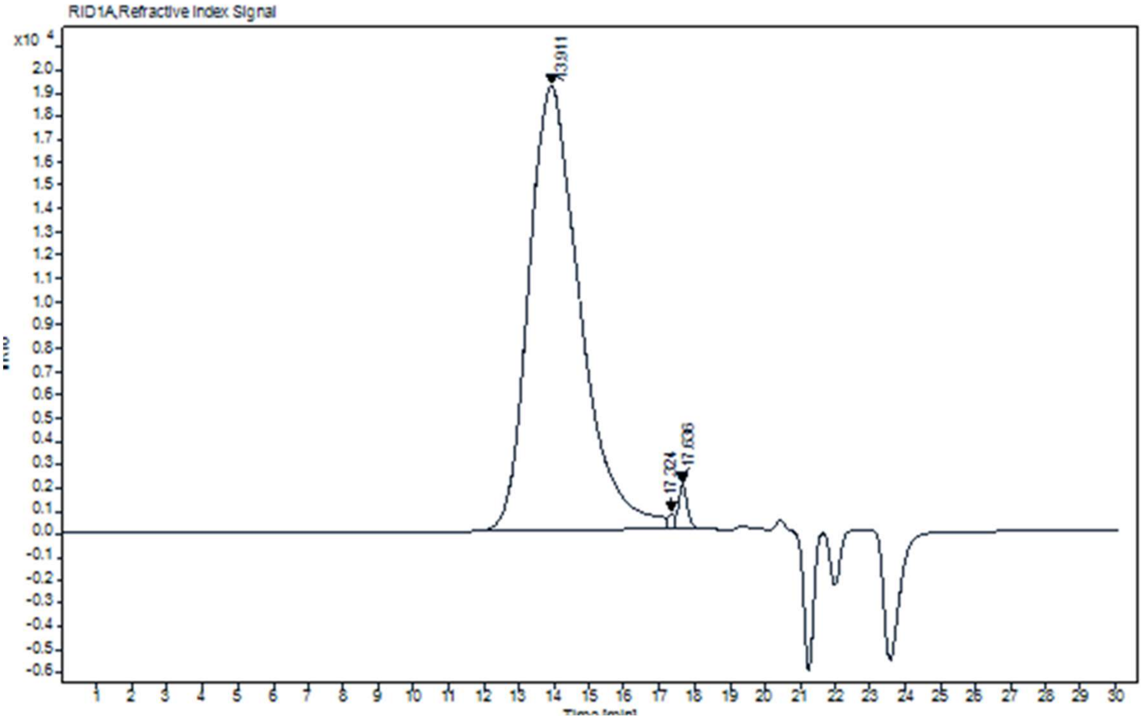

Figure S14. GPC results of P23B(58)ET

**Instrument:**	SP GPC	**Location:**	P1-E2
**Injection date:**	2023-10-20 15:56:11+02:00	**Injection:**	1 of 1
**Acq. method:**	GPC_DCM.amx	**Injection volume:**	50.000
**Analysis method:**	GPC_Standard processing method new calibration 11-Jan-	**Acq. operator:**	SYSTEM
**Last changed:**	2023-01-11 15:55:11+01:00		

Peak #	RT [min]	Mn [g/mol]	Mw [g/mol]	PD	Mp [g/mol]
1	14.137	18074	38747	2.14	40329
2	17.648	939	971	1.03	895

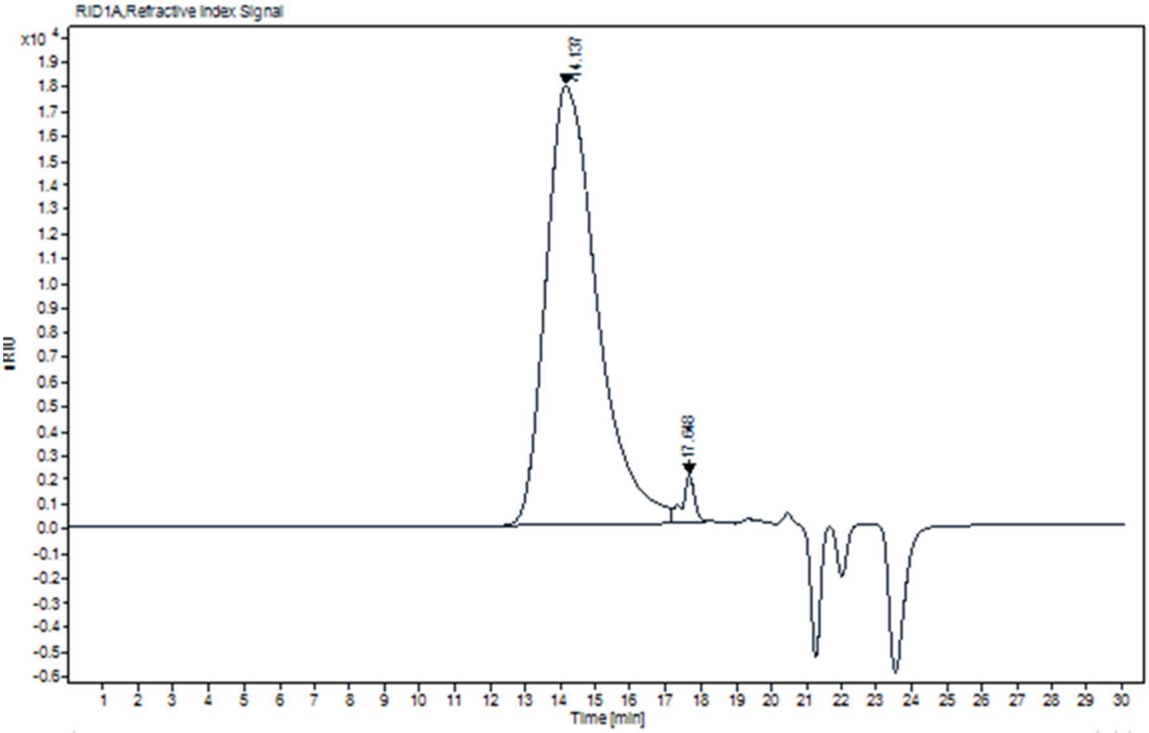

Figure S15. GPC results of P23B(78)ET

**Instrument:**	SP GPC	**Location:**	P1-E1
**Injection date:**	2023-10-20 13:29:29+02:00	**Injection:**	1 of 1
**Acq. method:**	GPC_DCM.amx	**Injection volume:**	50.000
**Analysis method:**	GPC_Standard processing method new calibration 11-Jan-23.pmx	**Acq. operator:**	SYSTEM
**Last changed:**	2023-01-11 15:55:11+01:00		

Peak #	RT (min)	Mn (g/mol)	Mw (g/mol)	PD	Mp (g/mol)
1	14.806	9794	18516	1.89	18528
2	17.301	1247	1253	1	1246
3	17.612	892	904	1.01	931
4	18.293	485	493	1.02	504

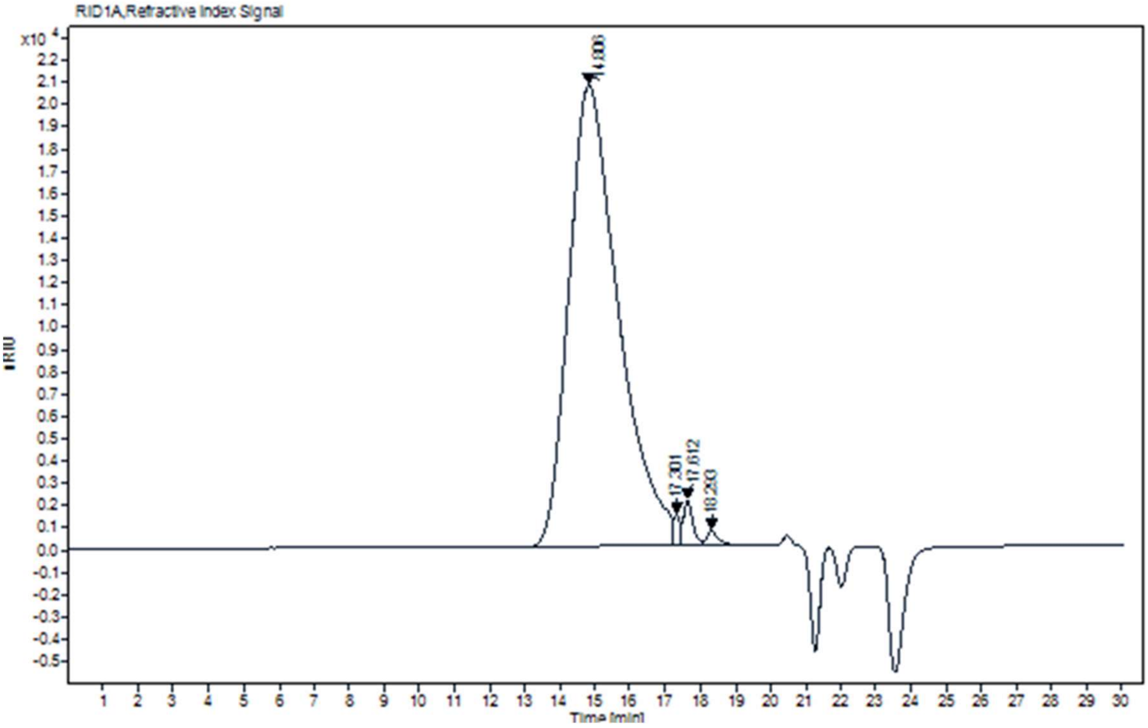

Figure S16. GPC results of P23BT

**Instrument:**	SP GPC	**Location:**	P1-D1
**Injection date:**	2024-03-27 11:30:31+01:00	**Injection:**	1 of 1
**Acq. method:**	GPC_DCM.amx	**Injection volume:**	50.000
**Analysis method:**	GPC_Standard processing method new calibration 11-Jan-23.pmx	**Acq. operator:**	SYSTEM
**Last changed:**	2023-01-11 15:55:11+01:00		

Peak #	RT (min)	Mn (g/mol)	Mw (g/mol)	PD	Mp (g/mol)
1	13.974	24080	50506	2.1	48811
2	17.363	1200	1205	1	1172
3	17.67	852	865	1.02	877

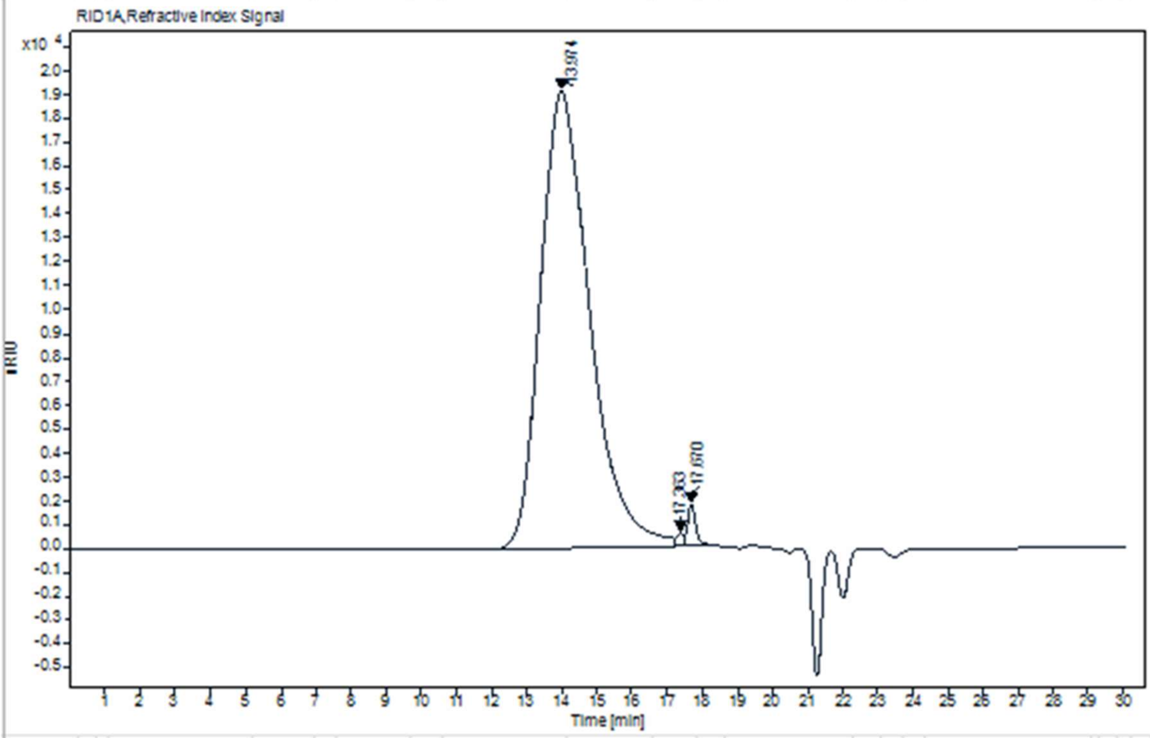

Figure S17. GPC results of P23B(43)ET

Impact test results

Table S8. Data impact tests

hammer 5 kpcm	
Sample **PET**	[J]
PET_0	0.123
PET_1	0.118
PET_2	0.083
PET_3	0.087
PET_4	0.08
Average	0.0982
Stdv	0.020584
Sample **PETG**	[J]
PETG_1	0.125
PETG_2	0.128
PETG_3	0.125
PETG_4	0.119
PETG_5	0.121
Average	0.1236
Stdv	0.003578
Sample **P23B(43)ET**	[J]
MBL119_1	0.079
MBL119_2	0.064
MBL119_3	0.072
MBL119_4	0.065
MBL119_5	0.06
Average	0.068
Stdv	0.007517
